# Supplementary material for: Optimization of chromium (VI) reduction in aqueous solution using magnetic Fe3O4 sludge resulting from electrocoagulation process
Source: PLoS One. 2024 Dec 31;19(12):e0309607. doi: 10.1371/journal.pone.0309607 (PMC11687653; doi:10.1371/journal.pone.0309607)
Supplement: S1 Table — (DOCX) [file pone.0309607.s001.docx]

**Table S1.** Adsorption isotherm for electro-coagulated Fe_3_O_4_ sludge

| **Adsorption isotherms** | **Lineer form** |
| --- | --- |
| Langmuir | $\frac{C_{A}}{q_{A}}=\frac{1}{b_{A}Q_{m}}+\frac{C_{A}}{q_{m}}$ |
| Freundlich | $\mathrm{Log}\left( q_{A} \right)=\log\left( K_{F} \right)+\left( \frac{1}{n} \right)log(C_{A})$ |
| D-R | $\mathrm{Ln}q_{A}=\mathrm{Ln}q_{s}-K_{\mathrm{ad}}{[RTLn \left( 1+\frac{1}{C_{A}} \right)]}^{2}$ |
| *Where Freundlich isotherm parametres: K_F_ is the Freundlich adsorption capacity parameter (mg/g), 1/n is the intensity parameter. D-R isothrem parameters: qs is the theoretical saturation capacity (mg/g), Kaf is the isotherm consant (mol^2^/kJ^2^). Langmuir isotherm parameters: Q_m_ (mg/g) and K (L/mg) are Langmuir constants related to adsorption capacity and energy of adsortion, respectively. | |
