# Supplementary material for: Optimization of chromium (VI) reduction in aqueous solution using magnetic Fe3O4 sludge resulting from electrocoagulation process
Source: PLoS One. 2024 Dec 31;19(12):e0309607. doi: 10.1371/journal.pone.0309607 (PMC11687653; doi:10.1371/journal.pone.0309607)
Supplement: S2 Table — (DOCX) [file pone.0309607.s002.docx]

**Table S2.** Adsorption kinetics for electro-coagulated Fe_3_O_4_ sludge

| **Adsorption Kinetic Models** | **Lineer form** |
| --- | --- |
| Pseudo-First-Order | $\log\left( q_{e}-q_{t} \right)={log}_{q_{e}}-\frac{K_{1}}{2.303}t$ |
| Pseudo-Second-Order | $\frac{t}{q_{t}}=\frac{1}{K_{2}q_{e}^{2}}+\frac{1}{q_{e}}t$ |
| Intraparticle Diffusion | $q_{t}=k_{i}x\sqrt{t+C}$ |
| *Where, q_e_ is the amount of adsorbent material in equilibrium (mg/g), q_t_ is the capacity at the time t (mg/g/min), t is the contact time (min), K1 and K_2_ are the first and second order kinetic constant, respectively. | |
