# Supplementary material for: Optimization of chromium (VI) reduction in aqueous solution using magnetic Fe3O4 sludge resulting from electrocoagulation process
Source: PLoS One. 2024 Dec 31;19(12):e0309607. doi: 10.1371/journal.pone.0309607 (PMC11687653; doi:10.1371/journal.pone.0309607)
Supplement: Attachment — Submitted filename: Reviewer.doc [file pone.0309607.s003.doc]

This manuscript reported the “Optimization of chromium (VI) reduction in aqueous solution using magnetic Fe_3_O_4_ sludge resulting from electrocoagulation process”. The manuscript needs some alterations before one can take a final decision. This review manuscript is still far from Major Revision in its current status because of following reasons:

1. Check the page for typo errors (e.g., space, superscript, subscript, spelling problems) on individual lines, such as 214, 240, 252, and 332.
2. The author should add a clear figure caption for Figure 5 (A, B, C, D) in the revised manuscript.
3. In section 3.6, the author said that Cr (VI) was reduced to Cr(III) in aqueous solution using a magnetic Fe_3_O_4_ sludge catalyst, however the author provided no data or results for the Cr(III) formation. UV-Vis spectra, a solution picture, and XPS data for Cr(III) reduction must all be included in the revised manuscript.
4. In Section 3.9, the author describes how to remove and reuse Cr(VI) from absorbed Fe_3_O_4_ sludge.
5. It is possible to test an industrial sample for Cr(VI) reduction using this Fe_3_O_4_ catalyst.
6. Line 132: The author said that "zeta potential analyses were performed for the characterization of electro-coagulated sludge." However, no zeta potential data is included in the manuscript. The revised manuscript must include zeta potential data.
7. Additionally, a hysteresis loop spectrum could possibly be provided for magnetic property confirmation.
